# Note on Valerianate of Zinc

**Published:** 1845-06

**Authors:** William Procter


### RECORD OF MEDICAL SCIENCE.

*Note on Valerianate of Zinc.* By William Procter, Jr.—Having had occasion to prepare this salt several months ago, the inconvenience of distilling so bulky an article as valerian (which swells much by maceration) was found to be so great, that in order to supersede the necessity of placing the root in the still the following method was resorted to, viz. Four pounds of valerian in coarse powder were thrown into two gallons of hot water, in which had been dissolved four drams of caustic potassa; the whole was heated for a short time, and then allowed to macerate for twenty-four hours, and then displaced with water until the liquid passing possessed but little taste. The clear infusion, which was slightly acid to litmus paper, was saturated, and evaporated until reduced to two pints, and as much diluted sulphuric acid was added as would neutralize the quantity of alkali employed. By this means the valerianate of potassa formed in the infusion was decomposed, and its acid set free. The whole was then placed in a glass retort on a sand bath, the distillation conducted until the liquid passing over was but slightly acid to litmus. The liquid in the receiver consists of a saturated solution of valerianic acid, with globules of that acid floating on its surface.

The next step in the process consists in preparing the hydrated carbonate of zinc by precipitation from a solution of the pure sulphate, by carbonate of soda, and washing to remove the saline matter.

The valerianic solution is then placed in a suitable vessel, an excess of the hydrated carbonate added, and the mixture heated until effervescence ceases and the saturation is effected,—the liquid should then be filtered, evaporated and crystallized. If the process has been carefully conducted, the product will amount to two drams, or about thirty grains per pound.

The salt thus obtained is often coloured brownish, and if required, can be obtained white by re-dissolving, treating with charcoal, and again crystallizing. Since adopting this method, which yields a larger product than by saturating the distilled water with the hydrated carbonate, I have seen a similar suggestion in the *L'Abeille Medicale*, by M. Guilleminet Macors, Pharmacien of Lyons, who, however, employed the carbonate of potassa.

M. Rabourdin, Pharmacien at Orleans, employs the following process, viz.: About eleven pounds of valerian to be distilled with a sufficient quantity of water, containing three troy ounces of concentrated sulphuric acid. When thirty pints of product has been obtained by distillation, the volatile oil is separated and the liquid saturated with from 22 to 25 drams of carbonate of soda, then evaporated to one pint, a slight excess of sulphuric acid added, and then distilled. By this process M. Rabourdin obtained from 11 to 12 drams of valerianic acid—a much larger quantity than is usually obtained.

By whatever process made, there should always be sufficient water to hold all the valerianate in solution when the liquid is hot, else a portion may be lost in separating the excess of carbonate of zinc. The salt requires about fifty times its weight of water for solution.—*American Journal of Pharmacy.*

